# Preventing fatal accidents in construction through the management of barriers

**DOI:** 10.1016/j.heliyon.2023.e21715

**Published:** 2023-10-26

**Authors:** Urban Kjellén

**Affiliations:** Norwegian University of Science and Technology (NTNU), Department of Industrial Engineering and Technology Management, NO-7491, Trondheim, Norway

**Keywords:** Occupational safety, Safety performance indicator, Barrier management, Construction, Fatal accidents, Digitization

## Abstract

The research presented in this article addresses the construction industry's need for a real-time safety performance indicator for use in managing the risk of fatal accidents. It involves the development and testing of an indicator of the availability of barriers against fatal accidents when carrying out construction work. In designing the indicator, knowledge and experience from different fields have been utilized including classic barrier theory, barrier management in major-accident hazard industries, quality management, and safety performance measurement in construction. The main aim of the research has been to evaluate the barrier indicator in practical use. The evaluation is based on results from field tests of the barrier indicator at two construction sites and on data from fatal accidents in the construction industry. It provided support for the assumption in the design of the barrier indicator that a few types of hazards dominate the statistics of fatal accidents. The field tests demonstrated the usefulness of the barrier indicator, both in checking the status of the barriers in individual construction activities and in measuring the overall standard of barriers against fatal accidents on the construction site. The possibilities and limitations of the method are discussed based on general criteria for assessing safety performance indicators. Another aim has been to use the experiences from the field tests as input to the design of a method for barrier management through the phases of a construction project. This work resulted in a model that integrates the barrier indicator and underlying methods and tools into a barrier management system. There is significant potential for effective barrier management by integrating the indicator and associated methods and tools into the management systems in the construction industry utilizing digital technology.

## 1 Introduction

Construction work involves the use of large amounts of energy, which under certain conditions can lead to fatal consequences of occupational accidents. This fact is reflected in the accident statistics. EU statistics for 2019 show that the number of fatal accidents per 100,000 persons employed was more than three times higher in construction compared to manufacturing [1]. The Norwegian fatal accident statistics show similar results. In addition, the fatal accident rate has remained at about the same level from 2012 to 2021 despite initiatives during the same period by the parties of the Norwegian construction industry to reduce this rate [2,3]. Construction here includes general construction and special trade construction for buildings and civil engineering, installation, and completion.

Measurement of safety performance plays an important role in any Occupational Health and Safety (OHS) management system, cf. ISO 45001, §9–10 [4]. Application of safety performance indicators such as the Fatal Accident Rate (FAR) and the Lost Time Injury Frequency Rate (LTI-rate) requires reporting of individual incidents, aggregation of data on enough incidents to produce meaningful statistics, and calculation of indicator value [5]. This process will result in feedback with significant delay. For a medium-sized construction site of e.g., 100 employees and an LTI-rate around 20, a reporting periodicity of less than 12 months will be meaningless due to statistical fluctuations. The data will be obsolete for use in feedback control even for large construction sites. This is a consequence of the fact that construction is organized into projects and that work at an individual site is of limited duration. It also goes through subsequent sub-phases with frequent changes in manpower and methods of work. The FAR is even less suited than the LTI rate for use as a basis for feedback control at the individual construction site due to a much lower frequency of fatal accidents. There was about one fatal accident to every 500 non-fatal accidents in construction in the EU in 2019 [1].

The applied research presented in this paper is part of a cooperation between The Norwegian University of Science and Technology (NTNU) and representatives from the Norwegian construction industry to address these critical issues. The aim of the project is to develop new types of performance indicators for use in construction that are better suited for timely and valid feedback to the site management about safety performance than the lagging indicators such as the FAR and the LTI rate in use today.

The current research uses the availability of barriers against high-consequence accidents as a basis for the development of a new type of indicator for real-time measurement of safety performance. Research shows that a large majority of severe accidents in construction are preceded by deviations in the affected construction system or in the production process [6]. The purpose of barriers is to make the construction system more resilient against deviations by intervening in an accident sequence during production to eliminate or reduce the potentially harmful outcome. From a management control point of view, the complexity of the control problem is grossly reduced by focusing on the availability of a limited set of barriers rather than on deviations in the construction system and process in general.

Measurement of barrier availability in this research utilizes a limited set of data from a selection of work systems and processes on construction sites. It represents a focused complement to the comprehensive methods of measurement of the effectiveness of the safety management process at different levels of the organization [[7], [8], [9]]. An advantage of the barrier indicator is that the relationship between the measurement results and the risk of fatal accidents is more transparent.

The development of the barrier indicator utilizes the fact that a few types of central events account for most of the fatal accidents in construction. Each type of event is associated with a specific type of hazard, i.e., source of energy [6,10]. Examples of activities involving such hazards are heavy lifting, operation of heavy mobile machinery, working at height, and blasting. Workers in infrastructure projects are also exposed to natural hazards such as rock-fall and landslides. The term barrier is here used in the classical sense as a specific set of safety measures that intervene in the sequence of events of an accident to prevent or limit accidental loss [11,12].

Barrier management has shown rapid development during the last 40 years [[13], [14], [15]]. This development has been spearheaded by industries that manage processes with major accident potential. In each of these processes, a few well-defined types of energy sources are present, e.g., flammable, and explosive substances (oil and gas, Petro-chemical), or uranium (nuclear power). The energy model by Gibson, Haddon's strategies for the prevention of harm, and the principles of defense-in-depth by use of multiple barriers represent theoretical frameworks that have been instrumental in developments in barrier management [[16], [17], [18]]. Each industry integrates barriers against major accident hazards typical for the industry in its technology for the design of facilities. The design also includes instrumentation for monitoring barrier performance. This is measured in real-time, and the results are used to ensure adequate barrier availability [19,20].

Previous research on the measurement of barrier availability as a means of managing the risk of accidents in construction uses a broad definition of the barrier concept. This means that many different measures that may reduce the probability or consequence of accidents can be included under the concept of barrier [8,9,21]. The present research differs from existing literature by limiting the scope of barrier systems to coherent sets of system elements in a production system that provide types of barrier functions like those successfully applied in the major-accident hazard industries. The purpose of the barrier functions is to intervene in the energy flow in different stages of the accident sequence to prevent or reduce a loss [14]. The research also differs from earlier research by focusing on the prevention of a limited set of dominating accident types in construction with high-consequence potential. These limitations allow for a focused approach benefitting from the experiences of barrier management in major-accident hazard industries.

In construction, workers are to a much larger extent involved in the hands-on control of large amounts of energy than is the case in major-accident hazard industries. Technology-driven monitoring thus must be replaced by a strategy for safety performance measurement based on auditing of construction work. Fortunately, suitable methods that may be adapted to this application already exist [22,23].

### 1.1 This paper

The overall aim of the current research is to provide the construction industry with an opportunity to significantly reduce the risk of fatal accidents by applying methods and tools for effectively managing barriers against such loss.

The main aim of this paper is to evaluate the barrier indicator based on data from a field test and historical data from fatal accidents in construction. A further aim is to provide input to the design of a comprehensive approach to the management of barriers against fatal accidents, based on experiences from the tests of the indicator and underlying methods and tools.

## 2 Theory

### 2.1 Barrier theory

#### 2.1.1 Characteristics of barriers, barrier analysis

The barrier indicator is rooted in the energy model, describing how injury results from an unwanted energy transfer to the human body above the body-injury threshold [11,13,16]. In construction work, the energy flow may be the same as that under controlled conditions is essential for the execution of work. The term barrier is here reserved for a set of integrated system elements with a function that intervenes in the accident sequence to prevent the uncontrolled release of energy (or uncontrolled movements by persons in the direction of the energy source) or to prevent or reduce the unwanted energy transfer to the human body and consequential damage [12,17].

Classic barrier theory, as summarized above, relies on the laws of physics. It differs from more radical interpretations of barriers such as those including human, technical/physical, or organizational measures or combinations of these at different system levels that can reduce the probability and/or consequence of accidents [21,[24], [25], [26]]. Classic barrier theory was selected in the development of the barrier indicator because it allows for a more practical delimitation of the boundaries of barriers than the radical interpretation. There is also strong empirical evidence of the effectiveness of safety measures based on Haddon in preventing severe accidents, e.g., in road transportation [27].

**Fig. 1:**
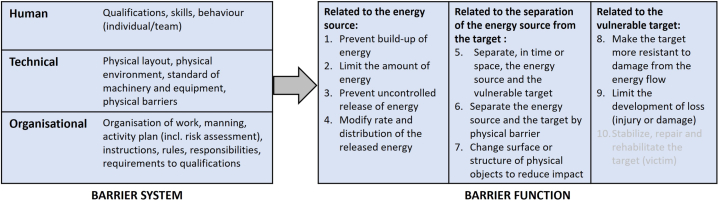
Barrier model applied in the development of the barrier indicator. The barrier system is illustrated by examples of barrier elements.

Fig. 1 shows the barrier model applied in the present work [12]. Nine of Haddon's “ten strategies for reducing damage from hazards of all kinds” have been used in the definition of barrier functions [17]. Strategy no. 10, “stabilize, repair, and rehabilitate the target of the hazard”, has been excluded since this type of measure lies outside the scope of the indicator. The barrier functions are performed by a barrier system, which may be purely technical or consist of integrated human, technical, and/or organizational barrier elements.

A barrier is either passive or active [12]. Passive barriers only include technical elements and are independent of any operational control system to execute the barrier function. A guardrail is an example of a passive barrier. An active barrier, on the other hand, is more complex and may consist of several human, technical, and organizational barrier elements. The barrier function is activated by a control system on the detection of a deviation or incident in an activity. An example of an active barrier is the braking system in a vehicle. This example also illustrates the fact that a system needed for normal production also may serve as a subsystem of a barrier system.

Barrier analysis has been a central method in the development of the barrier indicator [5]. It is used in accident investigations and risk analyses to identify the types of barriers needed from a defense-in-depth perspective to prevent a specified hazard from resulting in harm. The analysis is based on an analytic framework of the accident sequence with the loss-of-control of a hazard with fatal accident potential as the central event [24,28].

**Fig. 2:**
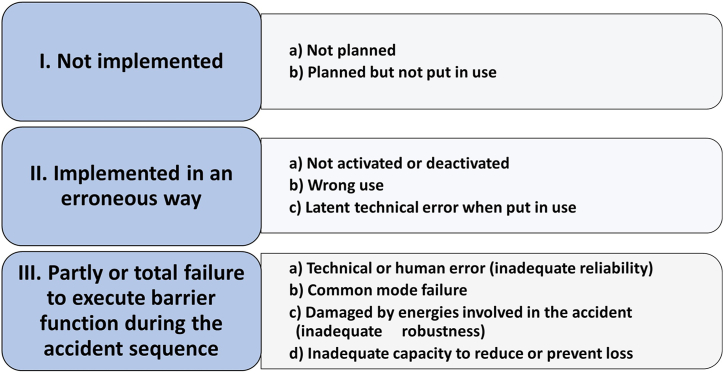
Limitations of barriers.

Barrier analysis also includes an analysis of the limitations of barriers, representing a further development of work by Trost and Nurtney [11]. Fig. 2 shows the classification of barrier limitations used in the work for this paper. The limitations have been identified on the bases of criteria for barrier performance, including the capacity to eliminate or reduce the unwanted energy transfer to the human body, reliability in delivering the barrier function when needed, and robustness to withstand accidental load [5,13].

#### 2.1.2 Barrier management

Barrier management is here referred to as the coordinated activities to establish and maintain safety barriers so that they achieve the organization's requirements for their functions to eliminate or reduce a loss [4,14,29]. In major accident hazard industry, it involves two distinct sets of activities [30]:

I. Planning and design: Identification of hazards and accident situations with the potential to cause harm. For hazards not eliminated in design, identification of the necessary barriers, barrier functions, and performance requirements to control the risk of loss.
II. Preparation for production and production: Implementation of required barriers, monitoring of performance, and maintenance of barriers.

Barrier management will affect several of the phases of a construction project and the cooperation between its different parties. The contracting process, and particularly the steps involving planning and design, tendering, award, mobilization, and production, are well suited to be used as a framework for the definition of tasks and responsibilities of these parties in barrier management [5,31].

### 2.2 Safety performance measurement

A safety performance indicator is here defined as a metric used in the measurement of an organization's effectiveness in controlling the risk of accidents in its activities [32]. It is used in feedback control of a production system through a cycle consisting of applying the indicator in the measurement of performance, comparison of the result with a norm for a desirable result, and implementation of corrective measures in case of a negative deviation from the norm [33,34].

The term ‘real-time indicator’ is here used for an indicator that provides results on safety performance with a short enough delay to allow for effective feedback control of the risk of accidents. They are generally based on observations and other types of measurements of the production process [5].

Behavioral sampling was developed in the 1950s as a safety performance indicator based on methods from statistical quality control of production to remedy critical shortcomings of traditional safety performance indicators such as the LTI rate [22]. This included timely feedback on performance results.

The work presented in this paper utilizes a more recent application of behavioral sampling, the TR method. It has been developed especially for the construction industry [23,35]. In the application of the method, independent inspectors observe behavior and physical conditions in the execution of construction work by use of a standardized checklist. Results are classified as either correct or not correct and are documented in a form that includes the checklist. The form is based on a template like that used by construction quality-control engineers. The TR index is calculated as a percentage of all valid observations that represent conformance with applicable safety requirements.

#### 2.2.1 Criteria for the assessment of safety performance indicators

Rockwell published a set of criteria for the assessment of safety performance indicators in connection with the work on behavioral sampling [22,32]. The criteria have been further developed and serve as a basis for evaluation in the present study [36]:

1. Flexible for use at different types of construction sites.
2. Address site conditions that are observable and quantifiable.
3. Valid measure of the risk of fatal accidents and sensitivity to change.
4. Transparent and easily understood by site personnel.
5. Produce reliable results when applied by different observers and robust against manipulation.

In the application of the TR method, reliability and robustness against manipulation are ensured using standardized and tested checklists and trained observers that are independent of the observation object and thus do not have a stake in the results [23,35].

The TR method has been subject to validity evaluation in two large-scale studies. Both tests used the LTI- rate (>2 days of absence) as a criterion variable [23,35]. The correlation turned out to be high.

The interobserver reliability of the TR method was checked and found adequate through tests involving parallel and simultaneous observations by different inspectors [23].

Another approach to validity assessment is to check whether a safety performance measurement system adequately measures what it is supposed to measure by comparing the contents of the measurement system with a generally accepted “gold” standard or as judged by a panel of recognized subject-matter experts. The former approach has been selected in content analyses of five different safety management audit systems used for improvements and benchmarking by applying a recognized OHS management standard as the “gold” standard [37]. Results were expressed as percentage compliance with the standard.

### 2.3 The barrier indicator

The barrier indicator is a real-time indicator based on audits of construction activities at individual sites. It represents a further development of safety performance measurement systems based on behavioral sampling and involves the application of classic barrier theory, Section 2.1.1.

The barrier indicator is primarily intended for use by the main actors of a construction project, the Client, and the Main contractor, as part of a comprehensive barrier management system. Use by the Main contractor will involve auditing of own and subcontractors' activities to investigate and follow up that applicable requirements to barriers are complied with. The Client may use the barrier indicator as a tool in system audits of the Main contractor's management of OHS at the construction site.

A construction project is a complex socio-technical system, where factors at different system levels affect the risk of fatal accidents [38]. The decision to develop a barrier indicator comprising the man-machine-technology (MTO) system at the work execution level with a direct effect on the fatality risk was made for reasons of user-friendliness and practicality.

Data collection and analysis cover relevant parts of each studied construction activity's MTO (man-technology-organization) system. This includes the personnel at the sharp end executing the activity, the machinery, tools, and materials in use, and applicable plans, instructions, and rules. It also includes corresponding MTO systems for simultaneous activities in the danger zone of the hazards involved in the audited activity where relevant.

In applying the barrier indicator, the primary audit objects are construction activities involving one or more of seven specified types of hazards. These hazards were selected based on an accident concentration analysis of fatal accidents in construction in Norway during 2011–2016. The analysis resulted in the identification of seven hazards that accounted for 78 % of the fatalities in the period [36]. These included:

1. Fall from or through a roof or deck
2. Fall from machine or equipment
3. Fall from an inclined or free-standing ladder
4. Hit by a falling load or another object in connection with lifting
5. Squeezed by crane or personnel lift in motion
6. Hit by a construction machine
7. Construction machine tips over or departs accidently from the road or work area

For each type of hazard, relevant barriers functions and associated barriers and barrier elements were identified based on barrier analyses. Data input to the analyses included accident statistics and in-depth investigations of fatal construction accidents, observations and interviews at construction sites, and scrutiny of regulatory requirements and lifesaving rules by major construction contractors. The results were analyzed to identify checkpoints suited for the checklists. In the next round, the checklists were subject to assessments for relevance, completeness, and user friendliness by senior construction personnel, representing construction site management, regional safety representatives, and quality control engineers.

The standard of barriers and barrier elements are audited based on these checklists, one for each type of hazard. All checklists apply the same standard quality control checklist template used in construction with three alternative outcomes for each checkpoint, OK (the results of the check are adequate), Deviation (the results represent a deviation from applicable norms), or NA (the checkpoint is not applicable to the activity in question). There is also space for a description of the findings (deviations) on the rear side of the checklist. The barrier index is calculated as a percentage of the total number of relevant findings that are adequate. This approach is consistent with that of the TR method [23].

The applicable norms are specific for each site and include regulatory requirements, producers’ manuals, and specific requirements in the safety management systems of involved contractors. In the audits, the quality of the norms is not subject to assessment, for example, regarding the application of recognized human factors principles [38].

**Fig. 3:**
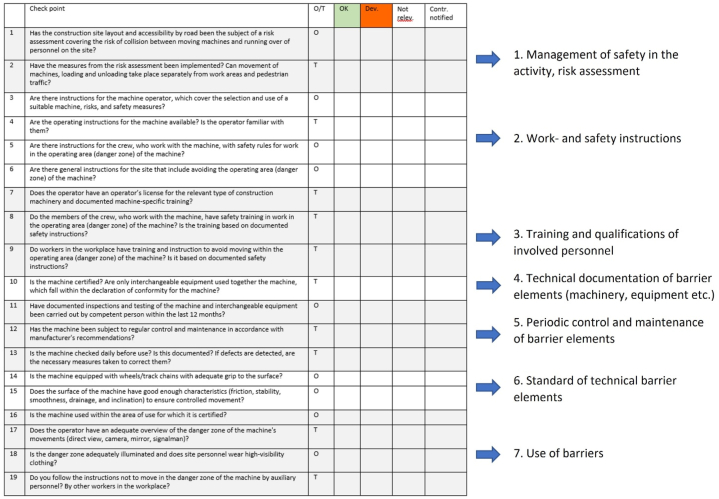
Checklist no. 6 and associated topics. Topic 8, “Emergency preparedness”, is not included in this checklist.

Checklist no. 6 in Fig. 3, “Hit by construction machine”, is here used as an example. It covers checking of barrier elements related to two barriers with different functions: Preventing uncontrolled release of energy (barrier function no. 3 in Fig. 1) and separating personnel from the danger zone of the machine when in motion (barrier function no. 5). The checklist is divided into topics, where Topic 1 is related to the management of barriers, and Topics 2–7 cover checkpoints related to the quality of individual barrier elements of relevance to the specified barriers. All seven checklists are structured in the same way.

Topic 1 aims at checking indirectly whether adequate measures have been taken in the planning and risk assessment of the construction activity. The topic has been included to cover site-specific conditions, not addressed under the more generic checkpoints below.

Topics 2 and 3 cover measures to secure adequate qualifications and skills for the personnel. Similarly, topics 4 and 5 address measures aimed at ensuring an adequate standard of machinery and equipment used in the work. Topic 6 addresses observable conditions of technical barrier elements, and topic 7 addresses the observable behavior of human “barrier elements”.

Checkpoints are specified as either “O" (observation), meaning that it is possible to answer the point based on an individual observation like in the TR method, and “T" (triangulation), indicating that the answer must be based on independent information sources. Checkpoint 17, the machine operator's overview of the danger zone, illustrates the need for triangulation based on observations of viewing conditions and personnel movements during work, interviews of the machine operator and ground personnel on their experiences, and review of relevant instructions.

Triangulation applies the principles of collection and verifying information in a management systems audit [39]. It follows that the users of the barrier indicator need to have adequate knowledge and skills in auditing. This includes the ability to conduct effective interviewing with due respect for the interviewee (i.e., “respectful dialogue”). It has been emphasized that the interview needs to be positive and nonconfrontational and address the current activity, why it is executed in the way it is, and the preconditions for relevant choices. Consequently, OHS advisors with a “policing” role perception will be less suited for the task because of the risk of blocking the interviewee from sharing relevant information.

## 3 Material and method

### 3.1 Overview of the research process

The starting point for the current research has been the lack of progress in the Norwegian construction industry in reducing the risk of fatal accidents. An idea of utilizing similar principles in preventing ordinary occupational accidents to those used by the Norwegian oil and gas industry in preventing major accidents was presented to the project steering committee and received acceptance [12].

**Fig. 4:**
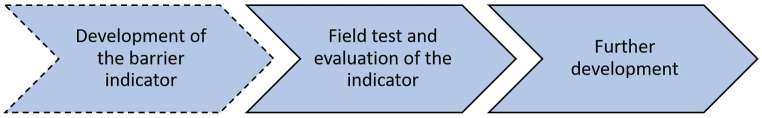
Research process. The focus of the present paper is on the two last phases.

Fig. 4 presents the research process for developing and testing the barrier indicator.

Development of the barrier indicator started in 2017 and resulted in user instructions and a description of the underlying theory [36,40]. Initial work was purely theoretical and involved the adaptation of barrier theory presented in Section 2.1.1 to the principles for activity management in construction. Further work included an analysis of the concentrations of fatal accidents in construction in Norway during 2011–2016, constituting 60 accidents with 63 fatalities. It turned out that about 70 % of the accidents belonged to one of three main categories: fall from height; hit or crushed by construction machine or vehicle in motion; or hit by load or equipment during material handling. Seven subcategories of the three main categories of accidents were identified and subsequently subject to further analysis. The purpose was to identify barriers for each subcategory with the ability to prevent or reduce harm. Each barrier was thereafter subject to an MTO analysis to identify human, technical, and organizational requirements for barrier elements in the construction system that needed to be met for the barrier to perform according to expectations. Results were summarized in checklists, one for each subcategory of accidents. These results were further reviewed and refined in workshops by high-level safety experts from construction client companies, contractors, and regional construction safety representatives. This work resulted in the final version applied in the field tests, see Section 2.3.

Two large state-owned construction client companies were initially approached with requests to provide construction sites for field tests. The intention was to carry out several field tests in parallel, involving both the construction client and the main contractor. Initial tests showed that sites had to be selected where the safety supervisor, responsible for applying the method, took an auditor role rather than a policing role in the application of the method. For this reason, the field test was limited to two construction sites belonging to the same Client company. Both sites involved tunnel rehabilitation.

The Main contractor participated in the test at the first site in parallel with the Client. Results from this part of the tests turned out to be unreliable and are not presented here. The difficulties in achieving construction contractor participation may be explained by the fact that the original commitment to offer construction sites for the tests was made by a construction client company. The contractor management of the affected projects lacked the necessary motivation to mobilize resources for increased control of own and subcontractor activities when this was not part of the contract with the Client company.

The pandemic during 2020–2022 also had negative impacts on the availability of construction sites for field tests and follow-up of results.

The objective of the field tests was to provide safety performance and experience data to be able to evaluate the barrier indicator method (cf. Section 2.2.1). Historic data on fatal accidents was also introduced in the evaluation of the indicator. It included the following activities:

1. Assessment of the representativeness of the selection of hazards that were included in the barrier indicator by comparing the original distribution of fatal accidents in construction in Norway in the period 2011–2016 with a corresponding distribution of fatal accidents in 2017–2021.
2. A content analysis of the results of the audits in the field tests based on the underlying barrier theory. To assess content validity, the results were compared with a corresponding analysis of results of in-depth investigations of fatal accidents in construction representing the same types of hazards as the barrier indicator.
3. To assess how transparent and comprehensible the indicator was for the involved site personnel, experiences from the use of the indicator were documented and followed up with interviews.

The analysis of the field test results identified the need for further development work beyond the original scope of the research to develop and test a barrier indicator, Fig. 4. It was executed by the project group in close cooperation with management and OHS staff at the client company involved in the field test. The work was based on the theories and principles behind the barrier indicator and on the principles of barrier management, Section 2.1.2.

### 3.2 Sources of data and specific methods

The Norwegian Labour Inspection Authority (NILA) compiles data on fatal accidents in the Norwegian construction industry. The authority receives reports on fatal accidents from the employer, police, or health services, and the reports are judged to represent close to 100 % of the actual number of fatalities in construction [6]. In the present study, data from NILA for 2017–2021 was received on a spreadsheet with basic information about accident type, type of work operation, type of injury, and a short description of the sequence of events. The data was scrutinized, and in some cases completed with information from other sources before each accident was classified with respect to type of hazard (energy source) and construction activity involved. The same classification schedule was used for the 2011–2016 data.

An accident concentration analysis played an important role in the original selection of construction hazards to be included in the barrier indicator [5,36]. The analysis utilized data from NILA on fatal accidents at Norwegian construction sites from the period 2011–2016. To check the stability of the distribution used in the selection, the analysis was repeated using data from the same source for 2017–2021.

Two projects involving upgrades of tunnels to the EU tunnel safety standard participated in the *field test* of the indicator. The manning of each project peaked at about 50 construction workers. An OHS/quality advisor from the Client's project organization executed the field tests for both projects. This included planning, data collection through interviews, observations, and document reviews and documentation of the results in the checklists. The documentation served as input to the analysis of the field test results. The author participated as an observer in the audits of about half of the activities involved in the field test. Data on experiences with the use of the barrier indicator were collected through interviews by the author with participants during and after completion of the test.

The five in-depth investigation reports used in the assessment of the content validity of the barrier indicator represented a “convenience sample” of reports of fatal accidents in construction involving any of the seven types of hazards of the indicator. The sample was taken from four infrastructure projects on three continents and involving different regulatory regimes. It was limited by the project team's access to relevant in-depth accident investigations where barrier analysis had been carried out. Recognized accident investigation experts had been involved in the investigations, and the results represent their assessments of findings on causes of barrier failures that are essential to the fatal outcome.

The results of the field test and the reports from in-depth accident investigations were subject to *content analyses* based on barrier analysis (cf. Section 2.1) and the checklists of the barrier indicator. For the accidents, this involves a stepwise analysis of:

1. Type of hazard involved,
2. Identification of relevant barrier functions and their performance (Fig. 1),
3. For barriers that failed, identification of barrier limitations (Fig. 2), and
4. Deviations in barrier elements (based on topics covered by the checklists, Fig. 3).

## 4 Results

### 4.1 Stability of the distribution of fatal accident concentrations used in the design of the barrier indicator

**Fig. 5:**
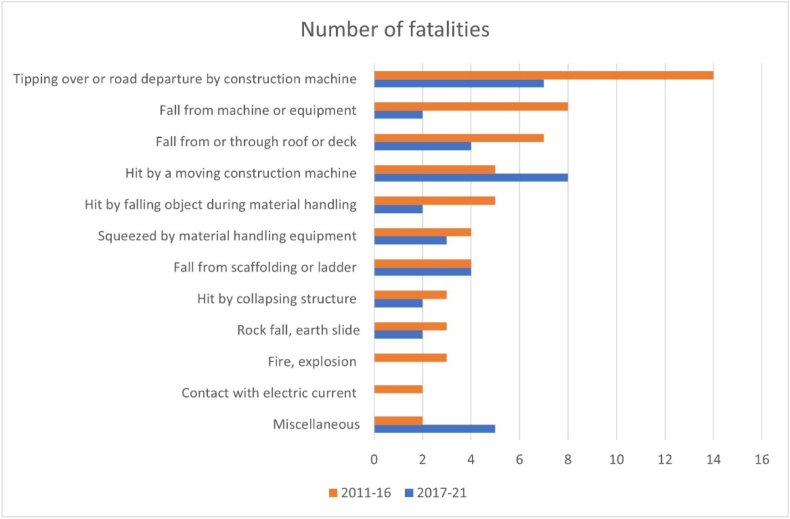
Number of fatalities in accidents in Construction 2011–2016 (N = 63) and 2017–2021 (N = 39) by type of hazard.

Fig. 5 shows the results of the analysis of fatal accident statistics from the NLIA's database for two periods, 2011–2016 and 2017–2021. The seven types of hazards included in the checklists for the barrier indicator accounted for 78 % of the fatalities in the first period. The corresponding number for the second period is 77 %, implying that the basis for the selection of hazards is reasonably stable.

We also note that there have been changes in the order of the dominating types of hazards in the fatality statistics. The “top three” in the 2011–2016 statistics included: “Tipping over or road departure by construction machine” (14 fatalities), “Fall from machine or equipment” (8), and “Fall from or through roof or deck” (7). For the 2017–2021 statistics, the corresponding types of hazards were: “Hit by moving construction machine” (10 fatalities), “Tipping over or road departure by construction machine” (9), “Fall from or through roof or deck”/“Fall from scaffolding or ladder” (4 each).

In the development of checklists, it was decided to focus on ladders rather than on scaffolds. The decision was made because there were three ladder fatalities and one scaffold fatality in the data of the first period. In the 2017–2021 statistics, the ratio between ladder and scaffold fatalities was one to three. The decision would have been different if based on statistics from the later period.

### 4.2 Field tests

#### 4.2.1 Results of auditing of construction activities

Table 1 summarizes the results of the field test by the Client in the two projects. In all, twelve construction activities were audited, and all had been selected from the progress plan of the Main contractors. Six of the seven checklists were tested in these audits, the exception was checklist no. 3, “fall from ladder”.

**Table 1:** Overview of the results of the field test in two projects by the Client, involving the collection of data on twelve different construction activities. For deviations, both the total number and the number of these that are made up of missing documentation are shown (the latter in italics).

Checklist no. (Section 2.3)	Number of activities	Number of checked items:				
		OK	Deviations:		Not applicable	Total
			All	*Inadequate documentation*		
1	1	7	5	*5*	3	15
2	4	24	8	*7*	28	60
3	0					0
4	1	12	3	*3*	2	17
5	2	15	8	*7*	3	26
6	2	25	10	*8*	3	38
7	2	17	10	*10*	13	40
SUM	12	100	44	*40*	52	196

In all, 144 checklist items were checked. Of these, 100 checks showed satisfactory results, and 44 checks were classified as deviations. This gives a barrier index of 69 %.

A large majority (91 %) of the identified deviations involved inadequate documentation. This result is in line with the important role of document checks in the checklists, which is required where observations and interviews are insufficient to judge whether barrier elements are adequate or not.

The four deviations not related to inadequate documentation were based on observable physical conditions and results of interviews and included missing fall-arrest system, missing cordoning off, and disrespect for the danger zone of machines.

In the first project, the OHS/quality advisor from the Client's organization and a safety engineer from the Main contractor conducted barrier index audits in parallel. The results from the Main contractor proved to be unreliable and are not presented here.

Below is a summary of typical findings in an analysis of the distribution of identified deviations by checklist topic, Table 2:

1. Management of safety, risk assessment: Lacking job safety analysis for the activity; contractor's risk analysis does not implement the Client's OHS plan; inadequate implementation of measures from risk analyses.
2. Work and safety instructions: Missing work or safety instructions; operating instructions for machinery missing or not available in the local language.
3. Qualifications of involved personnel (education and training): Education and training not documented for work at height; machine-specific training lacking; lack of documented and approved training for foreign nationals executing specialized work on a short-term basis (recurring topic)
4. Technical documentation of machinery and equipment: Lacking Declaration of Conformity for assembly of machinery (excavator with specialized tool); inadequate marking of equipment.
5. Periodic control and maintenance of barrier elements: Lack of documentation of regular maintenance of construction machinery, daily checks, and regular inspection/testing by a competent person.
6. Standard of technical barrier elements: Danger zone not roped off; automatic crash guard lacking on personnel lift.
7. Use of barriers: Fall arrest not in use; danger zone of heavy machinery not respected.
8. Emergency preparedness: Work at height rescue plan not adequately documented and trained.

**Table 2:** Distribution of identified deviations by checklist topic and checklist number.

Checklist topic (Fig. 3):	Checklist No. (Section 2.3):							SUM
	1	2	3	4	5	6	7	
1. Management of safety, risk assessment		2				1		3
2. Work and safety instructions	2	2		1	1	4	2	12
3. Qualifications of involved personnel (education, training)		3		1	2	1	2	9
4. Technical documentation of machinery and equipment				1	4	2	2	9
5. Periodic control and maintenance of barrier elements	1						4	5
6. Standard of technical barrier elements					1	1		2
7. Use of barriers		1				1		2
8. Emergency preparedness	2							2
SUM	5	8	0	3	8	10	10	44

#### 4.2.2 Comparison of field test results with results of barrier analyses in in-depth accident investigations

A compilation of the results of in-depth investigations of barrier performance in five fatal accidents is shown in Table 3. The results are consistent with the assumptions made in the design of the barrier indicator that a fatal accident within its scope requires the failure of a minimum of two barriers from providing their barrier function [36]. This includes failure of barrier function no. 3, “prevent uncontrolled release of energy”. The fatal consequence would have been avoided in all five accidents if one of the two barriers had performed as planned.

**Table 3:** Barrier analysis of five fatal accidents involving hazards covered by barrier index checklists no. 2, 4, 6, and 7.

Checklist no. (cf. Section 2.3) and accidental event	Consequence of failed barrier function (cf. Fig. 1)	Barrier limitations (cf. Fig. 2)
2. Fall from truss	3 Slipping and falling	IIa Moving outside designated access and work area
	2 & 4 (dual barrier function) Fall to the ground	IIa Safety harness not secured to truss
4. Hit by crane load	3 Loss of load	IIb Wrong rigging method used
	5 Other crew in danger zone of falling load	IIa Danger zone not evacuated, lifting over other crew
6. Squeezed between machine and tunnel wall	3 Uncontrolled movements of the machine on an uneven horizontal surface	IIc Uneven surface caused uncontrolled machine movements
	5 A crew member servicing a machine while occupying the danger zone	Ia Tacit acceptance of risk involving a violation of instructions in the machine manual not to occupy the danger zone
6. Trench worker hit by skidding machine	3 Uncontrolled skidding of the machine down a slope towards the trench	Ib Use of a machine not suited for the work
	5 Trenching worker in the danger zone of a skidding machine	Ib Departure from the plan of using an excavator for the work without a danger zone due to skidding hazard
7. Machine departed from road down a slope	3 Loss of control of machine movements	IIc Brake failure
	6 Machine continued through the roadside barrier and down a slope	IIc Space between roadside barriers allowed the machine to escape through
	6 Driver and passenger not protected inside the cabin	Ia Rollover protection system (incl. seat belts) not required

The accident reports included information on deviations in the barrier systems. This information has been categorized in accordance with the checklist topics (Fig. 3):

- Topic 1: Improvisation, ad hoc work involving bypassing of accepted work practice (three accidents), lack of planning by use of risk assessment and implementation (one accident)
- Topic 2: Incomplete lifting instructions (one accident), machine manual not available at the site (two accidents)
- Topic 3: Operator lacked machine operator license and machine-specific training (one accident)
- Topic 4: Use of old machine not subject to requirements to the documentation of fail-safe brakes and roll-over protection system (one accident)
- Topic 5: Machine not subject to regular maintenance and repair, brake failure experienced but not remedied (one accident)
- Topic 6: Uneven/slippery road surface (two accidents), track chains not suited for use in slippery slope (one accident), road-side barrier allowed the vehicle to pass through an opening in the barrier (one accident)
- Topic 7: Moving outside a designated area for work at height in combination with improper use of safety harness (one accident), use of wrong rigging tool for crane load in combination with lifting over an occupied area (one accident), use of an unsuitable machine for trench filling in combination with trench worker occupying the danger zone of the machine (one accident)

The importance of Topic 1 on barrier management is demonstrated by the results of this analysis. One fatal accident involved failure during planning to assess the risk of deactivation of a critical barrier. Improvisations in the execution of work by the work crew were present in three of the accidents.

A comparison of the results from the field test in the previous section with the analysis of the accident investigations shows notable differences in results within two of the eight checklist topics.

A critical result from the analysis of the accident investigations is the important role of improvisations in three of the five investigated accidents (Topic 1). It calls for a change in the sampling method used in the selection of construction activities to be subject to barrier indicator audits. The sampling must not be restricted to the main construction activities documented in the progress plan, as in the field test. Rather, they must constitute a representative sample of all construction and auxiliary activities at the site involving relevant hazards.

Topic 8 on emergency preparedness includes hazard-specific measures such as rescuing a person trapped at height. It was not an issue in the hazards represented by the investigations.

It is also notable that the investigations go more in-depth in checking the technical standard of the equipment (Topics 4 and 5), whereas the audit mainly stays short of that and relies on checking documentation and interviews with operators.

#### 4.2.3 Experiences from the field test

The Client's audits in the field test followed the same well-established routines as applied by the two projects' quality engineers in checking construction work. The general perception among the participants was that the checklists offered good support in checking individual activities for “holes” in barriers.

A major issue was the difficulty experienced by the client's OHS/quality advisor in getting access to documentation on checklist topics 1 to 5 from the Main contractor. This issue was not restricted to the test of the barrier indicator but was experienced as a more common issue facing the Client's OHS advisors. Experiences from the first field test showed that the Main contractor tacitly accepted lacking documentation from subcontractors of the qualifications of subcontractor personnel and technical standard of equipment. This condition was in interviews linked to a “trust climate” in the relations between the Main contractor and its subcontractors. Requesting such documentation from the subcontractors was seen as superfluous since the subcontractors had already been qualified for the work.

The usefulness of auditing short-term jobs was questioned, especially when there is a large flow of new short-term jobs, as there will be no time for correction before the job is completed.

### 4.3 Further development based on experience from the field tests

The initial scope of the current research was to develop and test a real-time indicator of barrier performance. Two experiences from the field tests pointed to the need to expand this scope by outlining a comprehensive system for barrier management system in a construction project (cf. Section 2.1.2).

The first experience was related to the extensive use of specialized construction workers on short-term assignments in tunnel rehabilitation projects. When barrier indicator audits were carried out of ongoing work, implementation of results would not have effect before work had already been completed. This applied, for example, to securing documentation of worker qualifications and the safety standard of equipment. The alternative was to stop work until such information had been secured. This would have cascading negative effects on cost and progress. The OHS advisor involved in the field tests suggested as remedial action to use the barrier indicator checklists for quality control of barrier status on two occasions, the first in the weekly planning meeting about two weeks before start-up, and the second in an audit immediately before start-up.

A second experience was illustrated by a case involving a physical barrier to prevent access to the danger zone during the pigging of the tunnel roof. The need for this barrier had been identified during project design and planning by the client's engineer and documented in the project's OHS plan. This was handed over to the Main contractor and implemented in the contractor's risk analysis for the project. It was deleted for reasons of practicability immediately before the start-up of work by the subcontractor and replaced by an active barrier involving instructions to the machine operator to watch out for trespassers. This barrier turned out to be unreliable since it was violated by construction personnel due to the need to cross the danger zone to make their way between two areas of the site in the lack of a safe alternative. The original physical barrier had never been adequately evaluated in the tender evaluation and contract negotiation phases or in the interaction between contractor and client after the contract award when the site layout was decided.

**Fig. 6:**
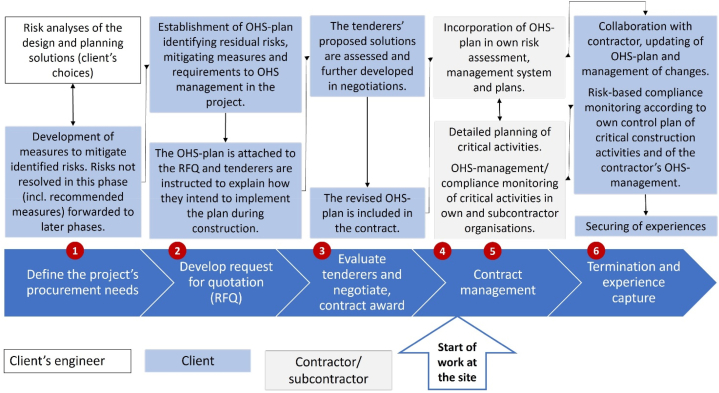
Risk management in the phases of the client's procurement process. Abbreviations: OHS = Occupational health and safety; RFQ = Request for quotation.

The project team took the Construction Client's Regulations as a point of departure in integrating a comprehensive risk management process into the Client's procurement process, Fig. 6 [41,42]. It involved definitions of the roles and responsibilities of the respective parties in this process.

Barrier management is an integrated part of risk management according to Fig. 6 (cf. Section 2.1.2):

1. Identification and assessment of hazards and barriers during design and planning may be accomplished by the combined use of risk analysis such as Job Safety Analysis and Barrier Analysis. It will focus on the consequences to safety of the architectural, technical, and organizational choices made in design and planning (cf. Construction Client's Regulations).
2. The OHS plan is founded in the Construction Client's Regulations and represents a carrier of experience through the different phases of the project. It is the Client's responsibility to ensure that the plan is kept updated and available to the different parties in the project.
3. Tenderers propose how to implement barriers to manage the risks identified in the OHS plan. These are evaluated by the Client as a basis for negotiations and the contract award.
4. Planning before the start of physical work at the site may include a collaboration phase between the Client and the Main Contractor, including joint risk and barrier analyses to quality assure activity plans and changes. Alternatively, this is done by the Main contractor and monitored by the Client. Results are included in the activity plans and in the quality and OHS control plans of the Main contractor and the client.
5. Implementation and quality assurance of required barrier elements at the planning stage and immediately before start-up of critical activities. Performance monitoring of the on-going activities includes application of the barrier indicator by the Main contractor and/or the Client.
6. The barrier indicator score of the project represents a measure of the compliance climate and may be captured by the Client for use in assessments of contractors in future projects.

This comprehensive scheme for barrier management was never tested in the current research project. Such testing must involve the procurement process and be reflected in the contracts between the project parties.

## 5 Discussion

### 5.1 General

The Norwegian construction industry has developed “life-saving rules” to prevent accidents due to hazards that dominate the fatal accident statistics [3]. Results presented in Section 4.1 indicate that such initiatives have been insufficient in changing the distribution of fatal accidents at the national level significantly. Neither do the national fatal accident statistics for construction indicate any significant improvement in the overall safety performance during 2012–2021 [2]. These statistical facts underline the need for new initiatives in construction safety management, and systematic barrier management offers such an opportunity.

### 5.2 Evaluation of the barrier indicator

The field tests have been less extensive than originally planned. They have covered two construction sites of similar characteristics and size, resulting in the audit of 17 activities by use of six of seven checklists. There has not been sufficient data to monitor the development of the barrier index over time. The test has not included documentation of how the findings from the application of the barrier indicator have been followed up.

Limitations in the execution of the field test mean that the results alone are not sufficient to allow for a comprehensive evaluation of the barrier indicator against the five criteria in Section 2.2.1. Hence, the theoretical and methodological context of the barrier indicator is referred to in the evaluation as well.

*Flexible for use at different types of construction sites (criterion no. 1):* Use of the barrier indicator produces two types of results: (1) a verification of the status of barriers against fatal accidents in individual construction activities, and (2) an index of the degree of compliance with regulatory and construction industry requirements to barriers at the site. The first type of results is relevant for all types of construction sites, where the construction hazards covered by the checklists are present. Size is critical for the second type of result. The sampling basis of construction activities covered by the checklists must not be too small when the results shall be used to monitor the development of the barrier index for the site in time. This limitation will affect the feasibility of using the barrier index as a safety performance indicator, depending on the size of the site, and the types of construction work carried out.

Individual construction sites may have different profiles of dominant fatal hazard types than the average of the industry. This applies, for example, to infrastructure projects, where hazards involving rockslides and explosives may play a more dominant role in the risk picture. Protection against such hazards is based on the same barrier principles as applied in the development of the current checklists. It follows that the barrier indicator may be adapted to the needs of the individual construction site.

*Address site conditions that are observable and quantifiable (no. 2):* This criterion has been focused on finetuning the checklists before the start of the field test. Quality control engineers were consulted in this work as they had the relevant know-how. Experience from the field test did not raise any critical issues regarding the quality of the checklists in this respect.

*A valid measure of the risk of fatal accidents and sensitivity to change (no. 3):* Ideally, work to validate the barrier indicator should be based on empirical testing with the fatal accident rate (FAR) as a criterion variable. This type of validation was used in the evaluation of the TR indicator with the LTI rate as the criterion variable, Section 2.2.1. It required massive testing on construction sites at a regional scale. The use of FAR as a criterion variable in validating the barrier indicator is not feasible since fatal accidents are much less frequent than lost-time injuries.

An assumption when designing the barrier indicator has been that a selection of dominant hazards is adequately representative of the total risk of fatal accidents. This assumption has been made to reduce the complexity of the indicator. A condition has been that the distribution of fatal accidents by type of hazard is stable over time. This condition was tested in the present study by comparing the original distribution of fatal accidents for 2011–2016 with a similar distribution for 2017–2021, Section 4.1. Results support the original assumption by showing that the distribution has been largely unchanged during the two periods in question.

The barrier indicator is based on recognized accident and barrier theory. The indicator measures compliance with specified quality criteria for individual barrier elements. These elements in combination form at least two barriers that simultaneously must fail for fatal loss to occur due to the specified hazard [36]. A score of 100 % on the barrier index does not guarantee fully satisfactory barrier performance when needed due to intrinsic variability in the performance of certain barrier elements. An operator constituting a human barrier element in a crane operation, for example, may make a mistake during the operation even if he/she meets all qualification criteria. Similarly, a technical element may fail even if it has been certified and subject to regular tests and maintenance. This residual uncertainty is compensated for by barrier redundancy. Simultaneous failure of two barriers scoring 100 % in the barrier index is regarded as unlikely.

The comparison in Section 4.2.2 between the results of the field tests with results on barrier analyses of in-depth accident investigation showed that the two activities gave comparable results. Although the sample of accident investigations for practical reasons has been very limited, the comparison is judged as a support of central assumptions in the design of the barrier indicator. These include the necessity of two simultaneous barrier failures to produce fatal loss, and the relevance and completeness of the eight topics included in the barrier indicator.

The analysis also illustrates the importance of auditing a representative sample of the construction activities at the site. It is thus not sufficient to select activities from the regular progress plan of the site as has been done in the test but must also include relevant ad-hoc activities not covered by the plan.

Two conditions in the development of the indicator also speak in favor of the assumption that the barrier indicator is valid as a measure of the risk of fatal accidents. First, the development of the indicator is based on a thorough review of regulations and industry standards representing the best available knowledge. The participation of senior safety experts and managers from construction client companies and contractors and regional safety representatives of the labor union in the development and review of the checklists contributes to improved validity as well.

All checkpoints in the checklists have been given equal weight in the calculation of the barrier index. Differentiation based on risk analysis would improve validity but would have certain negative effects like signaling to the organization that some requirements to barrier elements are less critical than others. It also makes the barrier index less easy to explain and comprehend and makes communication of causes of changes in performance results more complicated.

*Transparent and easily understood by site personnel (no. 4)*: The field test has not identified any significant concerns regarding this criterion. The checklists and methods for auditing have been subject to quality control based on this criterion during the development phase and are based on well-known principles and methods in use in the construction industry.

*Produce reliable results when applied by different observers and robust against manipulation (no. 5):* Due to the limited scope of the field test, there was no systematic check of the inter-observer reliability of the indicator. Reliability is both dependent on the quality of the checklists and on the qualifications and independence of auditors. Coordination of quality and OHS management in auditing of construction activities has the potential of improving access to qualified auditors and facilitating implementation of the barrier indicator.

The barrier indicator measures the degree of implementation and quality of concrete risk control measures. It is not exposed to the risk of manipulation as, for example, indicators of the activity level in safety management. Such indicators are vulnerable to management initiative to execute recordable activities requiring limited effort that inflate performance results but have uncertain effects on the prevention of accidents [8].

The vulnerability of the barrier indicator to manipulation due to insufficient independence of auditors, resulting in false assessment of audit results as OK, is a concern. It is a well-known vulnerability of safety performance measurement systems that depend on reporting by personnel with a stake in the results [5]. It is also the reason why independence from the auditee is required for auditors of management systems [39]. The TR indicator involved the use of independent safety inspectors in the observations for the same reason, Section 2.2. This vulnerability is critical since the barrier indicator is intended for use primarily by the Client and Main contractor organizations of a construction project. It must be carefully considered in the organization of the audits with respect to independence from the immediate line organization for production.

The field test did not include any systematic evaluation of the use of the information from the audits by site management. It has been assumed in the development of the indicator that construction organizations are accustomed to using the type of information produced in the application of the indicator as it is like the results from regular OHS practice.

### 5.3 Obstacles in implementing results and possible solutions

Experiences from the field test pointed at two obstacles in the implementation of results from the use of the barrier indicator. One was the short duration of some of the construction work at the sites, making it impossible to correct the identified deviations before this work was well underway or even completed. A second obstacle had primarily to do with conditions at the construction site that were decided without adequate consideration of potential conflicts with barrier availability and performance. Both obstacles initiated further development work to include the barrier indicator in a comprehensive system for barrier management, Section 4.3. Testing and validation of this solution will necessitate the implementation of barrier management in the contract between the Client and the Main Contractor.

A critical issue in the test of the barrier indicator has been the difficulties experienced by the auditor from the Client company to get access to relevant documentation on construction personnel and equipment. Digital technology offers a solution to this issue. It provides means for the effective acquisition and quality control of data and for analysis, storage, retrieval, and distribution. Such technology is to an increasing extent being taken into use by the construction industry [43,44]. A Norwegian example is “the Central Register” (sentralregisteret.no), which is a country-wide system for capture, quality assurance, and sharing of information on the competence of personnel and quality of machinery and equipment. If accepted for use by the Client and Main contractor, this solution is assumed to considerably reduce paperwork in applying the barrier indicator.

### 5.4 Potentials for further development

The present study demonstrates the value of analyzing combined data from OHS-activities such as accident investigations and auditing of safety-critical activities when the investigations and audits have a common theoretical and methodological basis. Machine learning technology is part of the development in digitalization of the construction industry and will provide new possibilities for the utilization of data from OHS activities. A generalized approach will involve use of an integrated system for the collection and use of different types of OHS data such as accident investigations, risk assessments, safety audits, safety performance measurement, and follow-up of deviations. It will open the application of dynamic risk modeling in decision-making in barrier management [19,45].

## 6 Conclusions

The risk of fatal accidents in the Norwegian construction industry remains a serious safety problem despite significant initiatives by the parties in the industry to reduce this risk. The development of the barrier indicator addresses a gap in the need for safety performance indicators to improve the management of this risk.

The indicator represents the use of a new and unique combination of already existing and proven theories and methods in the safety and quality management sciences. An evaluation based on experiences from field tests of the indicator did not identify any significant concerns in the applicability and usefulness of the barrier indicator in the management of high-consequence accident risks. There is a need for a further systematic collection of experience with the indicator and its integration into a comprehensive system of barrier management in construction projects. Experience from the field tests shows that this will be required to exploit the full potential of the barrier indicator. The barrier management system must cover early planning and design, contracting, and production, and needs to be specified in the contract between the Client and the Main contractor.

Implementation of systematic barrier management in construction must address “cultural issues”, including acceptance of the legitimacy of compliance checking at the site, and the ability of OHS advisors to engage in a respectful dialogue during audit activities.

Digitalization of the construction sector represents opportunities for radical simplifications in the implementation of the barrier indicator and in real-time feedback to decision-makers.

## Data availability statement

Has data associated with your study been deposited into a publicly available repository? No.

Why? Data included in article/supp. material/referenced in article.

## CRediT authorship contribution statement

**Urban Kjellén:** Writing – original draft.

## Declaration of competing interest

The author declares that he has no known competing financial interests or personal relationships that could have appeared to influence the work reported in this paper.
